# A hierarchical Bayesian Belief Network model of household water treatment behaviour in a suburban area: A case study of Palu—Indonesia

**DOI:** 10.1371/journal.pone.0241904

**Published:** 2020-11-06

**Authors:** D. Daniel, Mita Sirait, Saket Pande

**Affiliations:** 1 Department of Water Management, Delft University of Technology, Delft, The Netherlands; 2 Department of Ministry Quality and Impact, Health Units, Wahana Visi Indonesia, Tangerang Selatan, Indonesia; University of Vermont, UNITED STATES

## Abstract

Understanding the determinants of household water treatment (HWT) behavior in developing countries is important to increase the rate of its regular use so that households can have safe water at home. This is especially so when the quality of the water source is not reliable. We present a hierarchical Bayesian Belief Network (BBN) model supported by statistical analysis to explore the influence of household’s socio-economic characteristics (SECs) on the HWT behavior via household’s psychological factors. The model uses eight SECs, such as mother’s and father’s education, wealth, and religion, and five RANAS psychological factors, i.e., risk, attitude, norms, ability, and self-regulation to analyse HWT behavior in a suburban area in Palu, Indonesia. Structured household interviews were conducted among 202 households. We found that mother’s education is the most important SEC that influences the regular use of HWT. An educated mother has more positive attitude towards HWT and is more confident in her ability to perform HWT. Moreover, self-regulation, especially the attempt to deal with any barrier that hinders HWT practice, is the most important psychological factor that can change irregular HWT users to regular HWT users. Hence, this paper recommends to HWT-program implementers to identify potential barriers and discuss potential solutions with the target group in order to increase the probability of the target group being a regular HWT user.

## Introduction

The Sustainable Development Goals (SDGs) are more ambitious than the Millennium Development Goals because safety aspects of drinking water have been included as one of the new targets. Despite significant efforts to achieve this target in the past, three out of ten people worldwide still used contaminated water services in 2017 [1]. Since contaminated drinking water contributes significantly to water-related diseases, especially among the children below the age of five [2, 3], the safety aspect of the drinking water cannot ignored in efforts to achieve SDGs.

The trends of global drinking water service levels from 2000 to 2017 suggest that the target to have 100% safely managed drinking water services by 2030 is hard to achieve [1]. There is also a significant deterioration of water quality during transport and storage of water [4–7]. Therefore, it is important to have a more thoughtful, “interim,” approach in developing countries so that households can still consume safe drinking water.

Household water treatment (HWT), which means the use of any type of method to treat drinking water at a household level, such as boiling and water filtration, can be considered as an interim solution to improve the water quality at the household level even when the water quality from its source is contaminated [8, 9]. HWT can improve water quality and reduce water-related diseases, such as diarrhea, as long as the users perform it correctly and regularly [10]. However, HWT use has been declining [11] with many households performing HWT irregularly [12, 13].

The purpose of this study is to assess the regular practice of HWT among households in a suburban area of Palu, Province Central Sulawesi, Indonesia. According to the Demographic Health Survey in 2017, 66% of the total households in Central Sulawesi treated their drinking water, which is slightly below the national average 68% [14]. The HWT use in Central Sulawesi decrease slightly compared to the survey in 2012, i.e., 71%, while the national average remained constant [15]. We did not focus on a specific HWT method, but on general HWT behaviour. Therefore, “appropriate” HWT methods, i.e., boiling, water filtration, chlorination, and solar disinfection, were not differentiated.

A Bayesian Belief Network (BBN) model, which combines socio-economic characteristics (SEC) and psychological factors of households, is used to understand HWT behaviour. A three-level hierarchical BBN model is created with household’s socio-economic characteristics in the top layer, the psychological factors as the intermediate nodes, and the HWT behaviour as the output variable based on Daniel et al. [16], who found that the effect of SEC on HWT behaviour is mediated by the psychological factors. Finally, recommendations to increase the regular practice of HWT are also presented based on the obtained results.

## Methods

### Study setting

**Fig 1:**
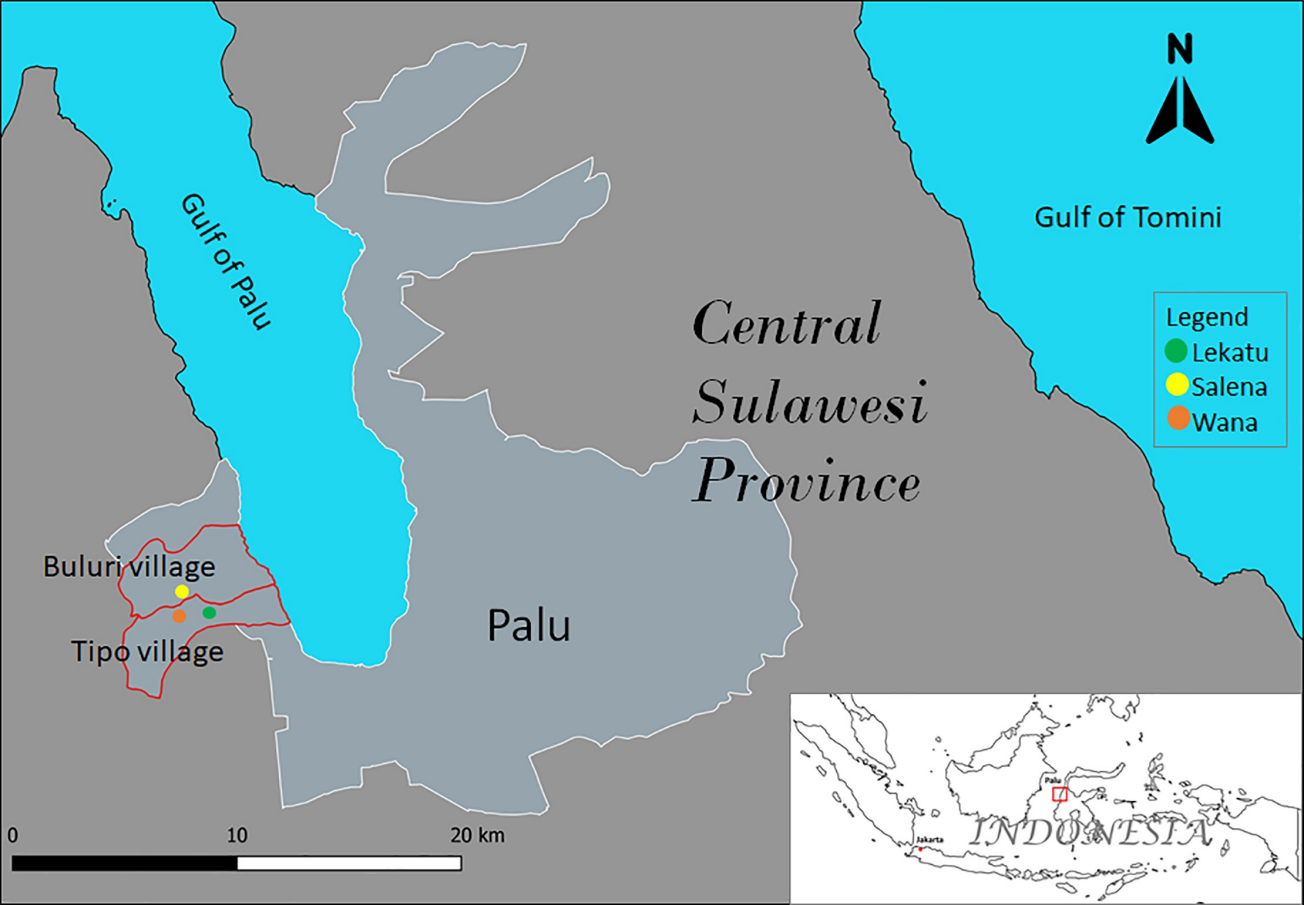
Map of the study locations.

We conducted the HWT behavioural study in July 2018 in the district of Palu, Province Central Sulawesi, Indonesia, in collaboration with a national NGO called Wahana Visi Indonesia (WVI). A total of 202 households were visited in three sub-villages within two suburban villages: (1) Wana and (2) Lekatu in village Tipo, and sub-village Salena in village Buluri (Fig 1). The sample size was obtained based in the methodology of [17] (check supporting information S1 for more information). These locations were selected as representatives of suburban villages of the *iReach* project, initiated and conducted by WVI, that have with high levels of diarrhea occurrence among children under the age of five years. The *iReach* project itself aims to improve the health of mothers and children in the district of Palu. Considering that many households in this project area still drink unsafe–untreated water, the surveys aimed to assess the practice and perceptions about HWT among the community.

We used a structured household interview which comprised of household’s socio-economic characteristics (SEC) information, such as parent’s education level, religion, and a list of household assets, and also HWT related information on knowledge, perceptions (psychological), and use related behaviour. We used mainly a five-Likert scale answers for the psychological factors and categorical answers for the SECs.

Six locals were trained to conduct the interviews and a pilot test was conducted before the real data collection. We mainly targeted the mother or primary caregiver for the interview. All participants gave written informed consent before being interviewed. The present study conformed to the guidelines of the declaration of Helsinki in human subjects. The study was approved by the human research ethics committee of Delft University of Technology and received government approval at the district level as part of the WVI *iReach* project. The first and second authors are from Indonesia and, therefore, do not need special permits to conduct the study.

### Socio-economic characteristics (SECs)

Eight socio-economic characteristics mentioned by previous studies influencing the HWT or other WASH behaviour were used: (1) *water-related health problem* [18, 19], (2) *information access* [20, 21], (3) *mother* and (4) *father’s education* [19, 22–25] (5) *wealth level* [26, 27], (6) *religion* [28–30], (7) *accessibility* [24, 31], and (8) *access to water* [25]. An answer to the question “how often do you watch TV?” (frequency watching TV) was used to represent *information access*, especially to mass media [32, 33]. For variable *water-related health problem*, we used information on the incidence of diarrhea among children below the age of five in the preceding two weeks of the time of the visit.

### Psychological factors

We followed the RANAS psychological framework to analyse HWT-related perceptions [34]. RANAS stands for *Risk*, *Attitude*, *Norms*, *Ability*, and *Self-regulation*, five psychological factors which are believed to be responsible for individual behaviourial outcome. *Risk* represents one’s awareness and understanding of the behaviour. *Attitude* is related to the feeling towards the behaviour. *Norms* represent social pressure towards the behaviour. *Ability* indicates one’s confidence in his or her ability to perform the behaviour. Lastly, *Self-regulation* depicts individual attempts to self-monitor and plan the behaviour and deal with conflicting goals. To cover well all aspects of each factor, RANAS framework uses several questions at a sub-factor level (Table 1). See [34] for detail definition of all the sub-factors. RANAS has been used in many HWT or WASH-related behaviour, see for example [35–39]

**Table 1:** Descriptive statistics of psychosocial factors.

Psychosocial factors		Example question	Scale	M(SD)
**Risk**	Perceived vulnerability	How high do you feel is the risk that you will get diarrhea if you drink untreated water?	1–5	2.64 (1.49)
	Health knowledge	Now, I will present you some measures that may help to prevent diarrhea. Please tell me for each option if you feel it is suitable as a preventive measure.	1–5^a^	3.00 (0.88)
	Perceived severity (on a child)	Imagine your child below 5 years has diarrhea, how severe would be the impact on his life and development?	1–5	3.59 (1.08)
**Attitude**	Health benefit	How certain are you that always treating your water will prevent you from getting diarrhea?	1–5	2.97 (1.34)
	Affective belief (taste)	How much do you like the taste of treated water?	1–5	3.29 (1.45)
	Affective belief (enjoy)	How much do you enjoy the moment when you treat your water?	1–5	3.49 (1.22)
**Norm**	Descriptive	How many of your neighbours treat their water?	1–5	2.06 (0.75)
	Injunctive	People who are important to you, how do they think you should always treat your water before consumption?	1–5	2.80 (1.19)
	Personal	How strongly do you feel an obligation to yourself to always treat your water before consumption?	1–5	3.32 (1.53)
**Ability**	Confidence in performance	How certain are you that you will always be able to treat your drinking water before drinking?	1–5	3.12 (1.41)
	Confidence in recovering	Imagine that you have stopped treating your water for several days, how confident are you that you would restart treating your drinking water again)?	1–5	2.72 (1.56)
	Confidence in continuation	Imagine that you have much work to do. How confident are you that you can always treat your water?	1–5	2.59 (1.57)
**Self-regulation**	Action control	How much do you pay attention to the resources needed to treat the water?	1–5	3.09 (1.25)
	Remembering	Within the last 24 hours: How often did it happen that you intended to treat your water and then forgot to do so?	1–5	2.84 (1.58)
	Commitment	How important is it for you to treat the water?	1–5	3.35 (1.34)
	Barrier planning	Could you tell me how do you deal with the obstacles that hinder you to treat water?	0–1^b^	0.3 (0.46)

M = mean, SD = standard deviation.

^a^ For *health knowledge*, the scale was based on the number of correct answers given by the respondents

^b^ for *barrier planning*, 1 = has clear solution, 0 = no clear solution.

### Outcome variables: *Household water treatment (HWT) behaviour*

To assess the practice of HWT among the respondents, a self-reported answer of whether they treat their drinking water at the time of visit were combined with respondents’ answers to four questions related to their HWT behaviour. The four questions corresponded to the frequency of drinking raw water daily, percentage of water treated daily, habit of performing HWT, and intention to treat water. The intention behind combining multiple answers is one of the strategies to diminish the bias in self-reported behaviour, which may overestimate the actual behaviour [16, 40–42].

### Bayesian Belief Network (BBN)

A Bayesian Belief Network (BBN) is a directed acyclic graph showing a hypothetical causal relationship between causal variable (called “parent node” in BBN) and the affected variable (called “child node”) [43]. The causal graph or the BBN structure represents the qualitative aspect of BBN since the structure is often inspired by conceptual theories or frameworks or expert consensus [44]. The quantitative aspect of BBN is reflected by the Conditional Probability Tables (CPT), which measure the strength of relationship between parent and child nodes.

### Data analysis

Two main analyses were conducted: (1) statistical analysis: the regression analysis; and (2) the BBN analysis.

Before conducting those two main analyses, the PCA was used to create variables corresponding to the nodes of the developed BBN model. These included *wealth level*, the five RANAS factors *Risk*, *Attitude*, *Norms*, *Ability*, and *Self-regulation*, and the output variable *HWT behaviour*. The PCA for *wealth level* was performed to estimate a representative value of relative wealth index of a household based on the observations of household assets [45]. A similar approach was used for the five RANAS factors. Since psychological information was available at sub-factor level (Table 1), PCA was used to “reduce” the dimensionality (information or the number of variables in the analysis) and capture the dominant information of the five main RANAS factors. For example, there are three sub-factors of *Risk*: *perceived vulnerability*, *health knowledge*, and *perceived severity*. PCA was used on these three sub-factors to obtain one representative variable for *Risk*. The same applies to the other four RANAS factors. PCA was also used to create output variable *HWT behaviour* using five related questions discussed in the section “outcome variable”.

Forced-entry multivariate regression analysis was performed using all RANAS sub-factors (Table 1) as predictor variables and variable *HWT behaviour* as the outcome variable. All statistical analysis used IBM SPSS Statistics 25 (IBM Corp., Armonk, NY).

For the BBN analysis, continuous valued variables were discretised since discrete valued BBN model was used. All PCA outputs were discretized into three categories. For the *wealth level*, the respondents were discretized based on their PCA scores: poor (the lowest 40%), middle (the next 40%), and rich (the last 20%) [45, 46]. Three levels were also assigned for psychological factors: low (lowest one-third of scores, e.g., low *Risk*), moderate (one-third to two-thirds of the lowest scores, e.g., moderate *Risk*), and high (the remaining data). Finally, a similar approach for the *HWT behaviour* was used and three categories were created: “non-user”, “irregular user”, and “regular user”. All the discretised variables were then used in the BBN analysis.

The BBN model was developed using *Genie 2*.*2* (www.bayesfusion.com) software package. The software utilizes the expectation maximization (EM) algorithm to estimate the CPTs within the model [47]. The algorithm has proven to be effective in estimating the CPTs in case of incomplete data [48]. The model’s performance was assessed using the same software using a ten-fold cross-validation test. The Area Under the Curve (AUC) value of the Receiver Operating Characteristics (ROC) curve showed model’s performance. A value close to one indicates perfect prediction of the output variable (higher sensitivity and lower false positives) [49]. A sensitivity analysis was also performed to identify sensitive model parameters (entries of CPT). Furthermore, predictive or Bayesian inference was conducted to simulate the effect of specific SECs and psychological nodes on the output node. The most important nodes are the nodes with the highest ΔP_*HWT behaviour = regular*_, i.e., highest difference in the probability of *HWT behaviour* being “regular” between before situation (current situation without any update) and after updating a specific node situation. For example, node *accessibility* is updated to 100% “easy” and it is observed how it changes the probability of “regular” state of output node *HWT behaviour*. The same approach was conducted to all categories or levels in all SEC and psychological nodes one at a time and ΔP_*HWT behaviour = regular*_ is analysed to identify important nodes. In addition, the Chi-square test was also conducted to confirm the statistical relationship between two categorical variables and strengthen the analysis of the BBN.

## Results

### Socio-demographic characteristics of the respondents

The majority of the respondents had tap connection: 45.5% had access to tap water inside the dwelling, while 41.4% of respondents relied on a public tap. 16.8% of the respondents had no formal education, while 25.7% of the household heads were not attending formal education. Only 13.9% of the respondents had their own toilet, 65.8% used a shared toilet, and 20.3% of the respondents still practiced open defecation. 68.7% of the respondents stated that they had received HWT promotion in the past. The percentage of households with children below the age of five was 55% (range from 1–4 children). All those 55% households also reported diarrhea among their children in the last two weeks at the time of visit. The majority of the respondents said that boiling is the most often HWT method that they practiced (88%), while small portion used other methods (7%), such as solar disinfection or filtration, and 5% stated that they do not use HWT at all. From the self-reported answer, only 38.1% of the respondents said that they are treating water at that moment. Furthermore, based on the PCA results using other pieces information (see section outcome variable), 33.7% of the respondents were categorised as regular HWT users.

### Regression analysis

Table 2 shows the results of regression analysis using all RANAS sub-factors as predictors of HWT use. According to the results, *barrier planning* (a person’s attempts to overcome barriers; a sub-factor of *Self-regulation*) is the most statistically significant psychological sub-factor, followed by *affective belief (taste)* (perception about the taste of water; *Attitude*) and *action control* (a person’s attempts to self-monitor a behaviour; *Self-regulation*) (see β value in Table 2). All other sub-factors in *Self-regulation* factor are also significant, as well as *perceived vulnerability* (perception on probability to get water-related disease) and *health knowledge* (knowledge on preventive measures of water-related disease) (both are *Risk* sub-factors) and *confidence in recovering* (perception’s on own ability to recover from setbacks; in *Ability* factor).

**Table 2:** Regression analysis of all RANAS sub-factors of psychosocial factors on HWT practice.

Variables	B	SE B	β
***Risk***			
Perceived vulnerability	0.079	0.030	0.117**
Health knowledge	-0.022	0.042	-0.017*
Perceived severity on a child	-0.081	0.034	-0.086
***Attitude***			
Health benefit	0.045	0.037	0.061
Affective belief (taste)	0.126	0.035	0.184***
Affective belief (enjoy)	-0.028	0.041	-0.034
***Norm***			
Descriptive	0.025	0.051	0.019
Injunctive	0.024	0.034	0.028
Personal norm	0.015	0.035	0.021
***Ability***			
Confidence in performance	-0.004	0.035	-0.006
Confidence in recovering	0.091	0.044	0.141*
Confidence in continuation	0.011	0.039	0.016
***Self-regulation***			
Action control	0.139	0.039	0.170***
Remembering	0.103	0.031	0.161***
Commitment	0.080	0.038	0.104*
Barrier planning	0.498	0.111	0.230***

*p ≤ 0.05

**p ≤ 0.01

***p ≤ 0.001. Adjusted R^2^ = 0.836, N = 158 after households with missing information were removed by the regression analysis.

### The Bayesian Belief Network (BBN) model

**Fig 2:**
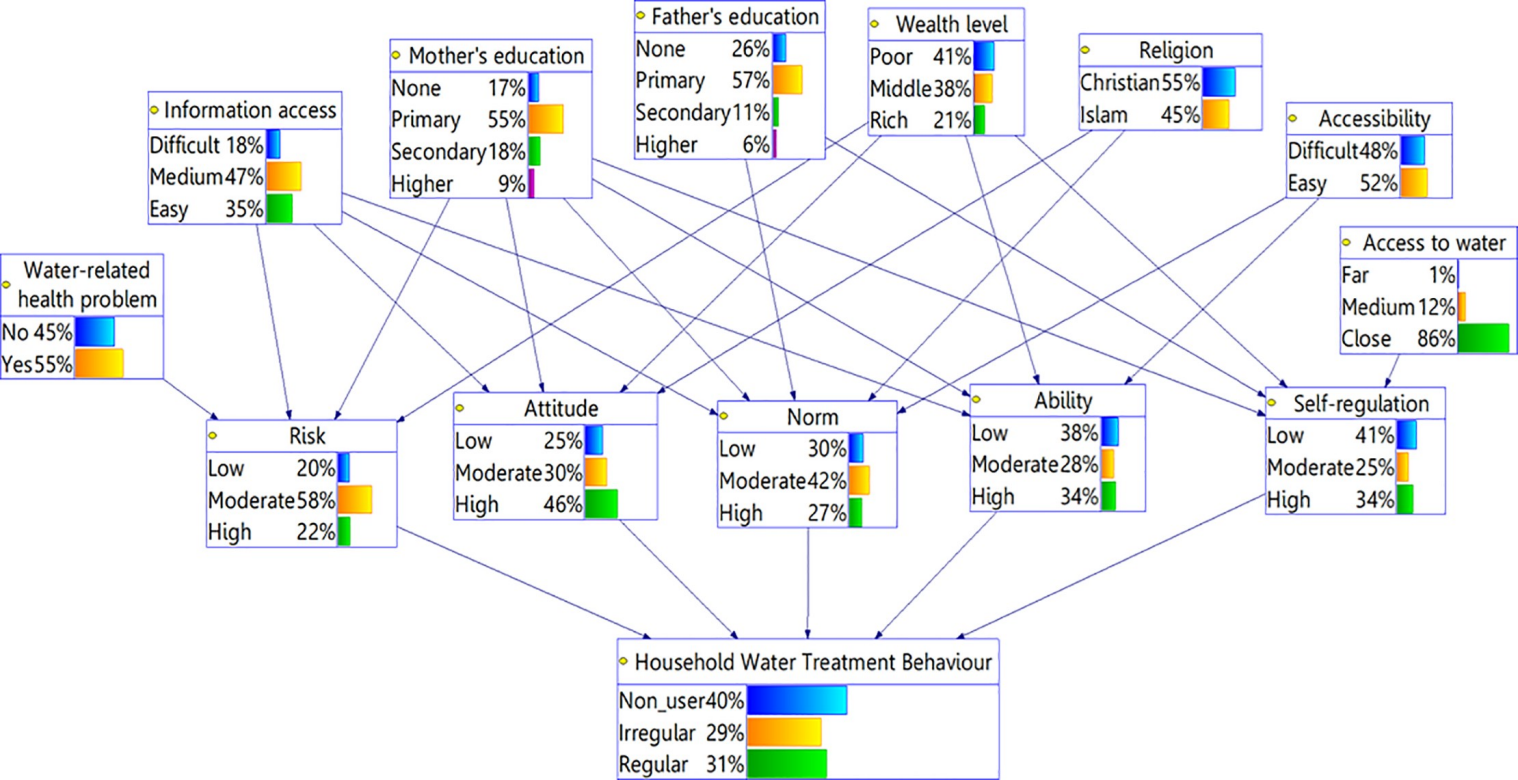
The hierarchical BBN model shows the hypothetical causal relationships between socio-economic characteristics (SEC), RANAS psychosocial factors, and HWT behaviour. The percentages in each node show the probability that a node is in a certain state. All 202 households were considered in calibrating the BBN model.

The BBN model is presented in Fig 2. It also shows the predicted probabilities of various states of the nodes after the model was calibrated (estimation of CPTs) on the household survey data. The model is “highly accurate”, according to Greiner et al., (2000), as shown by the AUC value of 0.90. The average model accuracy in predicting the output node is 79%.

**Fig 3:**
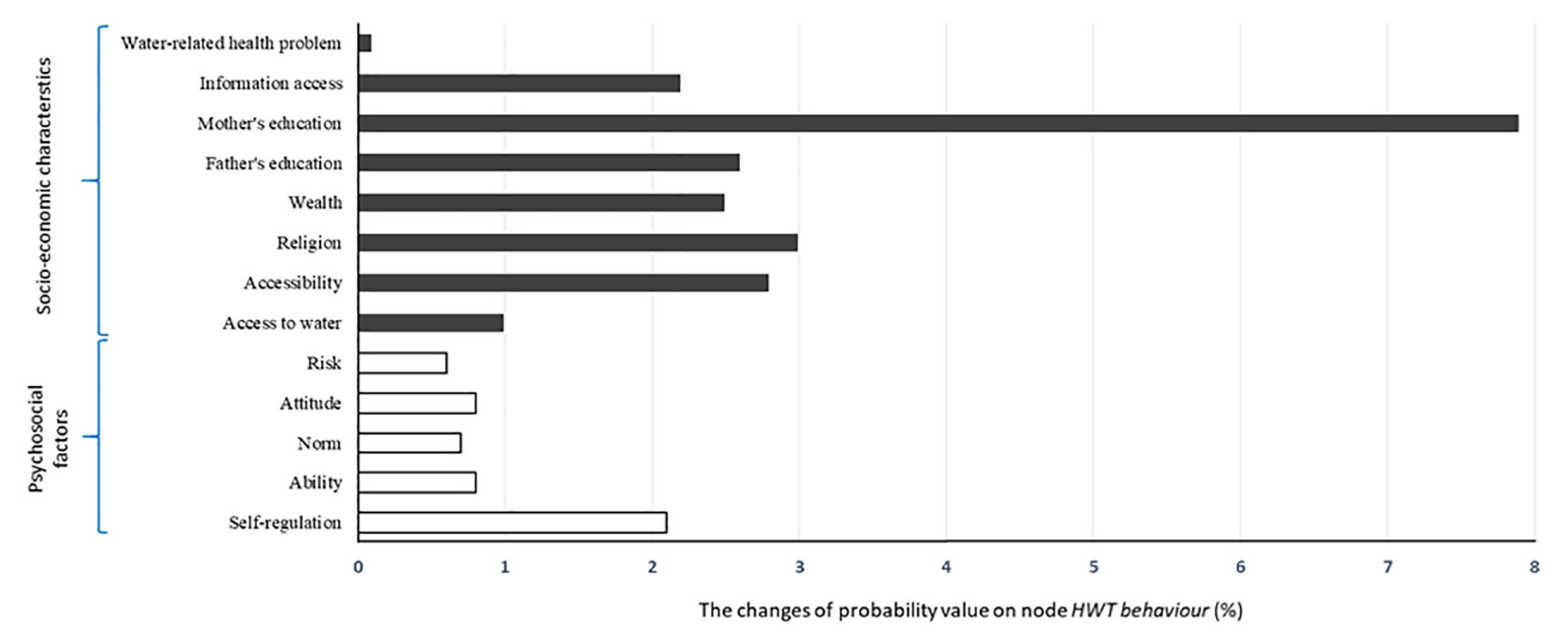
Sensitivity analysis of individual nodes on the probability of output node *HWT behaviour* being “regular”.

The sensitivity analysis shows that the node *mother’s education* is the most sensitive SEC and the node *self-regulation* is the most sensitive psychological factors (Fig 3). Node *Water-related health problem*, i.e., whether there was a diarrhea case in a household in the last two weeks, is far less important in the analysis. This implies that the occurrence of diarrhea among children below the age of five has no effect in influencing household’s psychology.

The predictive inference shows quite similar results with the sensitivity analysis, i.e., *mother’s education* and *self-regulation* are the most important SEC and psychological factor, respectively (Table 3). The influence of the node *mother’s education* is far bigger than any other SEC, i.e., ΔP_*HWT behaviour = regular*_ = 10%. When we looked at the influence of mother’s education on each of the five RANAS psychological factors in more detail, we found that the level of *attitude* and *ability* change quite significantly in response to a change in mother’s education compared to other psychological factors. Furthermore, the influence of each SEC node on the output variable was a “mixed” effect: better SEC does not always result in a higher probability of *HWT behaviour* being “regular”. For example, the higher the level of parent’s education and relatively easy access result in a higher probability of being “regular”, while access to water did not lead to regular use of HWT. There is a small effect of religion on the behaviour, even though not statistically significant (*X*^*2*^ (2) = 5.40, *p* = 0.07). In addition, there is small negative effect of wealth on the HWT behaviour, but this effect is low in BBN and far from significant in the statistical analysis (*X*^*2*^ (4) = 5.32, *p* = 0.26). Amongst the psychological nodes, *ability* comes up as the second most important node and *risk* is the least important node.

**Table 3:** Predictive inference that measures the effect of each state in each node on *HWT practice*. The value under each category corresponding to a node as displayed in the first column is the updated probability of the output node being “regular” given that all households maintain this state. The baseline probability was 31% (Fig 3).

Nodes		Updated P_HWT behaviour_ = regular (%) when probability of the node set as 100% to the state listed				ΔP_HWT behaviour_ = regular (%)^1^
**Socio-economic (SEC) characteristics**	Water-related health problem	No		Yes		0
		31		31		
	Information access	Difficult	Medium		Easy	2
		32	30		32	
	Mother's education	None	Primary	Secondary	Higher	10
		27	31	33	37	
	Father’s education	None	Primary	Secondary	Higher	2
		32	31	33	33	
	Wealth	Poor	Middle		Rich	3
		32	32		29	
	Religion	Christian		Islam		2
		32		30		
	Accessibility	Difficult		Easy		2
		30		32		
	Access water	Far	Medium		Close	0
		31	31		31	
**Psychological factors**	Risk	Low	Moderate		High	6
		31	30		36	
	Attitude	Low	Moderate		High	11
		28	26		37	
	Norm	Low	Moderate		High	10
		27	30		37	
	Ability	Low	Moderate		High	16
		25	28		41	
	Self-regulation	Low	Moderate		High	19
		23	31		42	

^1^The difference between the lowest and highest value of the updated probability of output node, *HWT behaviour* being “regular”, in %.

**Fig 4:**
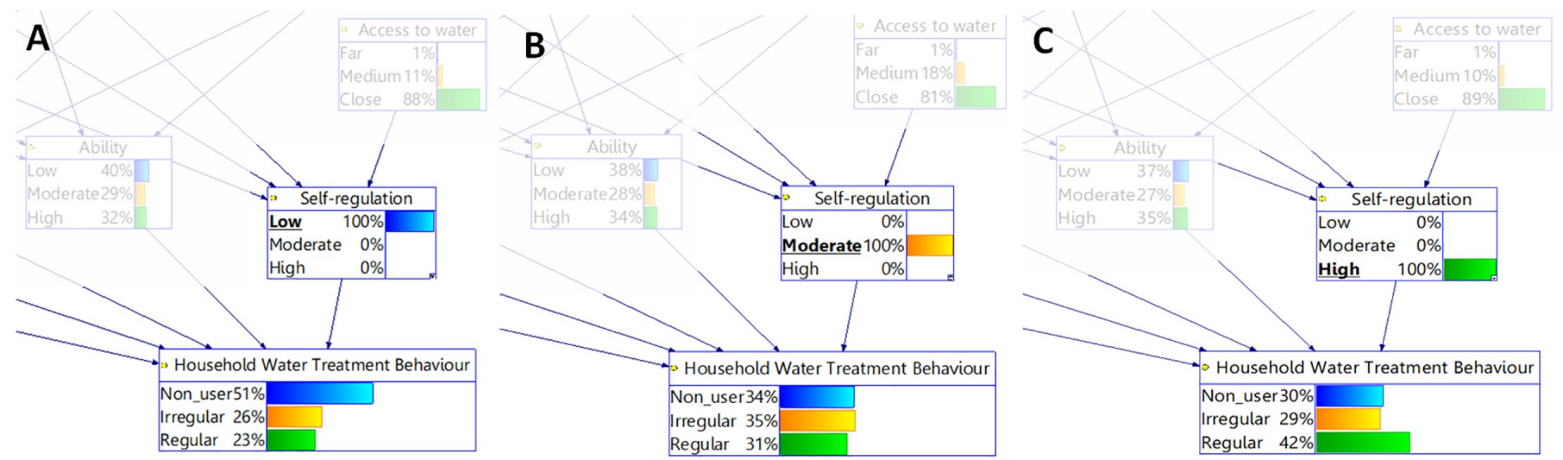
The variation of the probability at the output node *HWT behaviour* in response to the node *self-regulation* being in a certain state while holding the probabilities in all other nodes constant.

Since *self-regulation* is the most important psychological factor, how the output node *HWT behaviour* reacts to changes in probabilities in this node was investigated. Fig 4 shows that when the level of *self-regulation* is changed from 100% “low” to 100% “moderate” (compare Fig 4A and 4B), “non-user” group switches to “irregular user” group. The difference between “non-user” and “irregular user” probabilities is dramatic. The difference in probability of being a “non-user” and a “irregular user” in the situation of when the probability of *self-regulation* being “low” is 100% is 51–26% = 25% (Fig 4A). In comparison, the difference in probability of being a “non-user” and “irregular” user is 34–35% = 1% in the situation of “moderate” *self-regulation* (Fig 4B), when). The difference between “irregular user” and “regular user” probabilities was not high in the respective situations (26–23% = 3% in Fig 4A and 35–31% = 4% in the Fig 4B).

In contrast, when the state of *self-regulation* was changed from 100% “moderate” to 100% “high” (compare Fig 4B and 4C), “irregular user” group switched to “regular user.”The difference of probability of being a “irregular user” and a “regular user” in the situation of “moderate” *self-regulation* is 35–31% = 4% (Fig 4B). In comparison, the difference of probability of being a “irregular user” and a “regular user” in the situation of “high” *self-regulation* is 42–29% = 13% (Fig 4C). The difference in probability of being a “non-user” and a “irregular user” is not high in the respective situations.

## Discussion

Socio-economic characteristics of households are often considered as the root cause of any health-related behaviour [50, 51]. Moreover, since the influence of household’s SECs on the behaviour was found to be mediated by psychological factors [16], it is important to analyse them in one such causal system, wherein SECs of households can “influence” the psychology of households to use HWT regularly.

The sensitivity analysis and predictive inference suggest that the level of mother’s education is critical in identifying HWT users, i.e., whether they are non-user, irregular, or regular users. The influence of mother’s education on psychological factors *attitude* and *ability* are quite dominant. This implies that more educated mothers have more positive attitude towards HWT and have more confidence in their ability to perform HWT. Another interpretation is that targeting and educating mother, with regards to HWT or WASH issues, is an important step to change the community behaviour, especially because they are often responsible for managing water in the household [52]. In contrast to mother’s education, father’s education does not influence the HWT behaviour much, in contrast to Figueroa & Kincaid [25] who indicated that father’s education may influence the household’s norm.

The influence of other SECs is far less influential. For example, diarrhea occurrence among the children and access to water do not influence the psychology of households to adopt HWT much. Access to mass media, such as TV, and type of religion that households follow also have little influence on the behaviour.

*Self-regulation* appears to be the most important psychological factor. This in line with the results of statistical analysis in which all sub-factors of self-regulation are statistically significant (Table 2). There was significant decline in the percentage of non-users when the probability of *self-regulation* was changed from 100% “low” to 100% “moderate” and significant increase in the probability of regular users was observed when the probability of self-regulation was changed from 100% “moderate” to 100% “high”. This finding shows how psychosocial factor *self-regulation* changes the non-users to irregular users and also from irregular users to regular users. This suggests that self-regulation, i.e., self-monitoring and evaluating their own current behaviour, is critical to convert irregular users to regular users, as also has been suggested by [34]. Moreover, the sub-factor *barrier planning* of *self-regulation* comes up as the most significant sub-factor according to the regression analysis. This means that households that have strategies to overcome possible barriers that hinder the behaviour are more likely to practice HWT regularly.

Hence, suggested strategies to change the behaviour are, first, to discuss and make a list of possible barriers with the respondents and help them to come up with potential solutions or strategies to overcome those barriers. Afterward, the counsellor or implementer should encourage the respondents to apply those strategies, i.e., eliminating physical and social interferences that may bar them from adopting the behaviour and to anticipate other barriers [53].

The important limitation in this study is that since the study was conducted in the intervention locations of the ongoing project of the NGO WVI, the responses may suffer from social desirability bias. However, we tried to minimize it by explaining the anonymity and confidentiality of their responses and mentioning that the study is conducted by independent university which is not related to the NGO. Furthermore, even though the sample size was sufficient to identify important factors associated with the HWT behaviour [54], larger sample size may provide more solid interpretations. Finally, most of the respondents were familiar with boiling and we are aware that there are some concerns related to the practice of boiling, such as time or cost spent, type of fuel used, and the issue of household air pollution [55, 56]. These concerns are out of the scope of this study but need to be taken into account by the project officer.

## Conclusion

The socio-economic characteristics and psychological determinants of household water treatment behaviour in a suburban area of Indonesia were investigated using a Bayesian Belief Network model. The mother’s education level was the most important socioeconomic characteristics, while *self-regulation* was the most important psychological factor. Mother’s education influences the level of attitude and ability of households with regard to HWT behaviour. *Self-regulation* was found to be critical for the continuation of the HWT behaviour, i.e. change the irregular users to regular users. The self-regulation’s sub-factor *barrier planning* was found very significant on the behaviour. This suggests that households that are able to overcome potential barriers to perform HWT have a higher chance to perform HWT regularly.

## Supporting information

S1 Data (XLSX)

S1 File (XLSX)
